# Pyrroloquinoline Quinone Attenuates Traumatic Brain Injury‐Induced Secondary Damage by Activating PINK1/Parkin‐Mediated Mitophagy and Suppressing ASS1/CPS1‐Driven Arginine Biosynthesis

**DOI:** 10.1002/cns.71146

**Published:** 2026-09-09

**Authors:** Yanan He, Lu Yu, Yixun Lu, Zhikang Zhou, Ao Li, Lu Wang, Zhili Huang, Yanhong Liu, Yulong Ma, Weidong Mi

**Affiliations:** ^1^ Department of Anesthesiology The First Medical Center of Chinese PLA General Hospital Beijing China; ^2^ Beijing Key Laboratory of Perioperative Multimodal Digital Intelligence‐Integrated Diagnosis and Treatment Beijing China; ^3^ Department of Pharmacology, School of Basic Medical Sciences, State Key Laboratory of Medical Neurobiology Fudan University Shanghai China

**Keywords:** arginine biosynthesis, ASS1, CPS1, mitophagy, PINK1/parkin, pyrroloquinoline quinone, traumatic brain injury

## Abstract

**Aims:**

Pyrroloquinoline quinone (PQQ) was reported to be neuroprotective after experimental traumatic brain injury (TBI), but its mechanisms remain undefined. We tested whether PQQ protects against TBI in mice and identified the associated pathways.

**Methods:**

Male C57BL/6 mice received intraperitoneal PQQ (6.25, 12.5 or 25 mg/kg) immediately after controlled cortical impact. Mortality, modified neurological severity score (mNSS) and beam balance were followed to day 14; histopathology, immunofluorescence, western blot, ELISA and ATP assays were performed on day 3. Transcriptomics, metabolomics, network pharmacology and docking were integrated to identify candidate mechanisms.

**Results:**

PQQ reduced mortality (lowest at 12.5 mg/kg) and dose‐dependently improved neurological deficits, neuronal apoptosis, brain edema, pro‐inflammatory cytokines, and oxidative stress; the mNSS and beam balance benefits persisted to day 14. At 12.5 mg/kg, multi‐omics and network pharmacology identified arginine biosynthesis, mediated by argininosuccinate synthetase 1 (ASS1) and carbamoyl phosphate synthetase 1 (CPS1), as the top‐ranked pathway suppressed by PQQ; docking predicted binding of PQQ to both enzymes, suggesting putative targets pending validation. PQQ concurrently restored PTEN‐induced kinase 1 (PINK1)/Parkin‐mediated mitophagy and ATP production.

**Conclusion:**

PQQ attenuates secondary injury after TBI in male mice, in association with increased markers of PINK1/Parkin‐mediated mitophagy initiation and suppressed ASS1/CPS1‐driven arginine biosynthesis, identifying a candidate dual‐axis mechanism and nominating the mitophagy‐arginine axis as a target for neuroprotection in TBI.

## Graphical abstract

Pyrroloquinoline quinone (PQQ) attenuates traumatic brain injury in mice. Multi‐omics integration and molecular docking reveal that PQQ activates PINK1/Parkin‐mediated mitophagy to restore mitochondrial homeostasis while suppressing ASS1/CPS1‐driven arginine biosynthesis to dampen neuroinflammation, identifying a dual‐axis therapeutic mechanism for TBI.

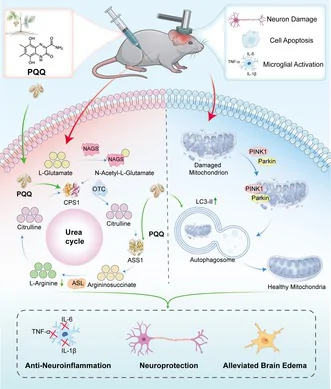

## 1 Introduction

Traumatic brain injury (TBI) is a leading cause of disability and mortality worldwide, with approximately 20.84 million incident cases and 5.48 million years lived with disability in 2021 [1], and is increasingly recognized not as an acute event but as a chronic disease carrying an elevated risk of late‐onset neurodegeneration [2]. Primary mechanical injury triggers a secondary cascade of neuroinflammation, oxidative stress, mitochondrial dysfunction, neuronal apoptosis, and cerebral edema [3] that drives progressive deterioration, and no pharmacological treatment has yet shown definitive clinical efficacy [4].

Mitochondrial dysfunction occupies a central position in this cascade. Reactive oxygen species (ROS) damage mitochondria, triggering PINK1 accumulation on the outer mitochondrial membrane, Parkin recruitment and selective mitophagy of damaged organelles [5]; the PINK1/Parkin pathway also regulates NOD‐like receptor protein 3 (NLRP3) inflammasome activation, linking mitochondrial quality control to neuroinflammation [6]. In parallel, metabolic reprogramming through the arginine biosynthesis pathway, particularly the urea‐cycle enzymes argininosuccinate synthetase 1 (ASS1) and carbamoyl phosphate synthetase 1 (CPS1), has emerged as a determinant of neuroinflammatory tone and neuronal survival after TBI [6]. Impaired mitophagy, excessive ROS, and disrupted arginine metabolism together amplify neuroinflammation and accelerate neuronal death, making them attractive therapeutic targets.

Pyrroloquinoline quinone (PQQ) is a redox‐active quinone occurring naturally in fermented foods, vegetables and human breast milk, with established roles in cellular energy metabolism, antioxidant defense and neuroprotection [7, 8]. Its reported mechanisms include activation of nuclear factor erythroid 2‐related factor 2 (Nrf2)/antioxidant response element signaling, enhancement of mitochondrial biogenesis through AMP‐activated protein kinase (AMPK)/peroxisome proliferator‐activated receptor γ coactivator‐1α (PGC‐1α), and suppression of nuclear factor‐κB signaling [9]. PQQ preserves neurocognitive function in rodent models of stroke and brain injury and attenuates α‐synuclein fibril formation and mitochondria‐related neurodegeneration [10, 11], and has been studied in lung injury, Alzheimer's disease and Parkinson's disease [12]. An early study reported that PQQ attenuates experimental TBI, but did not examine the molecular mechanisms involved [13]. Here we confirm that PQQ attenuates TBI‐induced neurological deficits and neuropathological damage in vivo and, building on that report, use multi‐omics profiling to show that the protection is accompanied by restoration of PINK1/Parkin‐mediated mitophagy and by suppression of ASS1/CPS1‐driven arginine biosynthesis.

## 2 Materials and Methods

### 2.1 Animals and Experimental Design

All animal procedures were approved by the IACUC of the First Medical Center of Chinese PLA General Hospital (No. 2026‐X23‐28), followed the NIH Guide, and are reported per ARRIVE 2.0. Male C57BL/6 mice (8–10 weeks, 22–25 g) were randomized by computer‐generated sequence to Sham, TBI, TBI + vehicle, and TBI + PQQ (6.25, 12.5, 25 mg/kg), with allocation concealed by coded cage cards; no formal power calculation was performed. Predefined exclusions (impactor missing preset depth/velocity, dural laceration, premature death) triggered replacement in tissue cohorts. Independent cohorts (per group): survival *n* = 24; behavior *n* = 6; histological staining *n* = 3; brain water content *n* = 6; ELISA *n* = 9; western blot *n* = 3; metabolomics *n* = 10; transcriptomics *n* = 5. PQQ (10% DMSO/40% PEG300/5% Tween‐80/45% saline) was administered as a single i.p. injection immediately post‐injury; controls received vehicle. Acquisition and quantification were blinded via coded labels; tissue was harvested on day 3. Based on published rodent dose ranges and an in‐house inverted‐U pilot, 12.5 mg/kg served as the mechanistic dose; the full range was retained for mortality and mNSS readouts.

### 2.2 Neurobehavioral Assessment

Neurological function was evaluated on days 1, 4, 7 and 14 post‐TBI using the modified Neurological Severity Score (mNSS; range 0–18, higher scores indicating greater impairment) in all five arms of the independent behavioral cohort (Sham, TBI and the three PQQ dose groups; *n* = 6 mice per group). Motor coordination and balance were assessed by the beam balance test on days 1, 7 and 14 in the Sham, TBI and TBI + PQQ (12.5 mg/kg) arms of the same cohort (*n* = 6 per group), graded on a 6‐point scale (0 = falls off; 6 = steady balance with all four paws); the mean of three trials per animal was recorded. All behavioral assessments were performed by two investigators blinded to group allocation.

### 2.3 Histological, Immunofluorescence and Western Blot Analyses

Perfused, paraffin‐embedded coronal sections (5 μm) were stained with hematoxylin‐eosin, NeuN, Nissl, and TUNEL to assess morphological damage, neuronal survival, cytological integrity, and apoptosis, respectively; representative low‐ (500 μm) and high‐magnification (50 μm) images were quantified in ImageJ, averaging three non‐adjacent sections per animal. Cryosections (10 μm) were immunostained for AQP‐4, GFAP, and IBA1 (astrocyte activation, blood–brain barrier integrity, microglial activation) and, for mitophagy, co‐stained for LC3 (red) and TOMM20 (green); confocal images (20 μm mitophagy, 50 μm others) were quantified in ImageJ as a percentage of Sham. For western blotting, perilesional tissue was lysed in RIPA buffer, resolved by SDS‐PAGE (30 μg/lane), and probed for AQP‐4, GFAP, IBA1, PINK1 (64 kDa), Parkin (52 kDa), COX IV (17 kDa), TOMM20 (16 kDa), LC3‐II/LC3‐I (14/16 kDa), ASS1 (45 kDa), CPS1 (150 kDa), and β‐actin (43 kDa); bands were detected by enhanced chemiluminescence and quantified relative to β‐actin. All acquisition and quantification were blinded.

### 2.4 Brain Water Content, ELISA, and ATP Measurement

Brain edema was quantified by the wet‐dry method: [(wet − dry weight)/wet weight] × 100. IL‐6, TNF‐α, IL‐1β, MDA, SOD, and GSH were measured in tissue homogenates by commercial ELISA kits (pg/mg, pg/mg, pg/mg, nmol/mg, ng/mg, and mmol/g protein, respectively). ATP was quantified by a bioluminescence kit (Servicebio, G4309) and expressed as μmol/kg tissue.

### 2.5 Transcriptomic Sequencing and Systems‐Level Analysis

Total RNA from perilesional brain tissue of the Sham, TBI and TBI + PQQ (12.5 mg/kg) groups (*n* = 5 per group) was sequenced on an Illumina NovaSeq platform. Reads were aligned to GRCm38 with HISAT2 and quantified with featureCounts; differentially expressed genes (DEGs) were identified with DESeq2 (|log_2_FC| > 1, adjusted *p < 0.05*), and GO and KEGG enrichment were performed with clusterProfiler. Two complementary systems‐level analyses were then applied to the DEGs. For the rescued‐gene analysis, log_2_ fold‐changes of TBI + PQQ versus TBI were plotted against TBI vs. Sham, and genes whose PQQ‐induced change opposed the TBI change were defined as PQQ‐rescued. Weighted gene co‐expression network analysis (WGCNA R package), restricted to the 1266 TBI‐DEGs given the five‐animal group size, built a signed network at a scale‐free soft‐thresholding power; modules were defined by dynamic tree cutting, eigengenes correlated with group, and hub genes identified by module membership (kME).

### 2.6 Metabolomic Profiling

Perilesional brain tissue from the Sham, TBI, and TBI + PQQ groups (*n* = 10 per group) was profiled by liquid chromatography–tandem mass spectrometry, and raw data were processed in XCMS. Differentially expressed metabolites were defined by variable importance in projection > 1 and *p < 0.05* from OPLS‐DA, followed by KEGG enrichment and hierarchical clustering. Each OPLS‐DA model was validated by sevenfold cross‐validation and 100‐iteration response permutation testing. Data quality was monitored with pooled QC samples, and unsupervised PCA was performed on all samples before downstream analysis.

### 2.7 Network Pharmacology and Molecular Docking

PQQ‐related targets from the CTD, TargetNet, and STITCH databases and TBI‐related targets from GeneCards and OMIM were intersected, and the shared targets were subjected to GO and KEGG enrichment in DAVID (false discovery rate < 0.05). A protein–protein interaction network was built in STRING (confidence > 0.7), and hub genes were defined by intersecting the top 10 genes from four CytoHubba algorithms. Molecular docking of PQQ to the corresponding RCSB PDB structures used AutoDock Vina 1.5.6.

### 2.8 Statistical Analysis

Each animal represents one biological replicate; technical replicates were averaged before analysis. Data are mean ± SD. Normality and homogeneity of variance were assessed by Shapiro–Wilk and Brown‐Forsythe tests; when met, groups were compared by one‐way ANOVA with Tukey's post hoc test; otherwise by Kruskal‐Wallis with Dunn's test. *p* < 0.05 was significant. Analyses used GraphPad Prism 9.0.

## 3 Results

### 3.1 PQQ Attenuates TBI‐Induced Neurological Deficits and Neuropathological Damage

**FIGURE 1:**
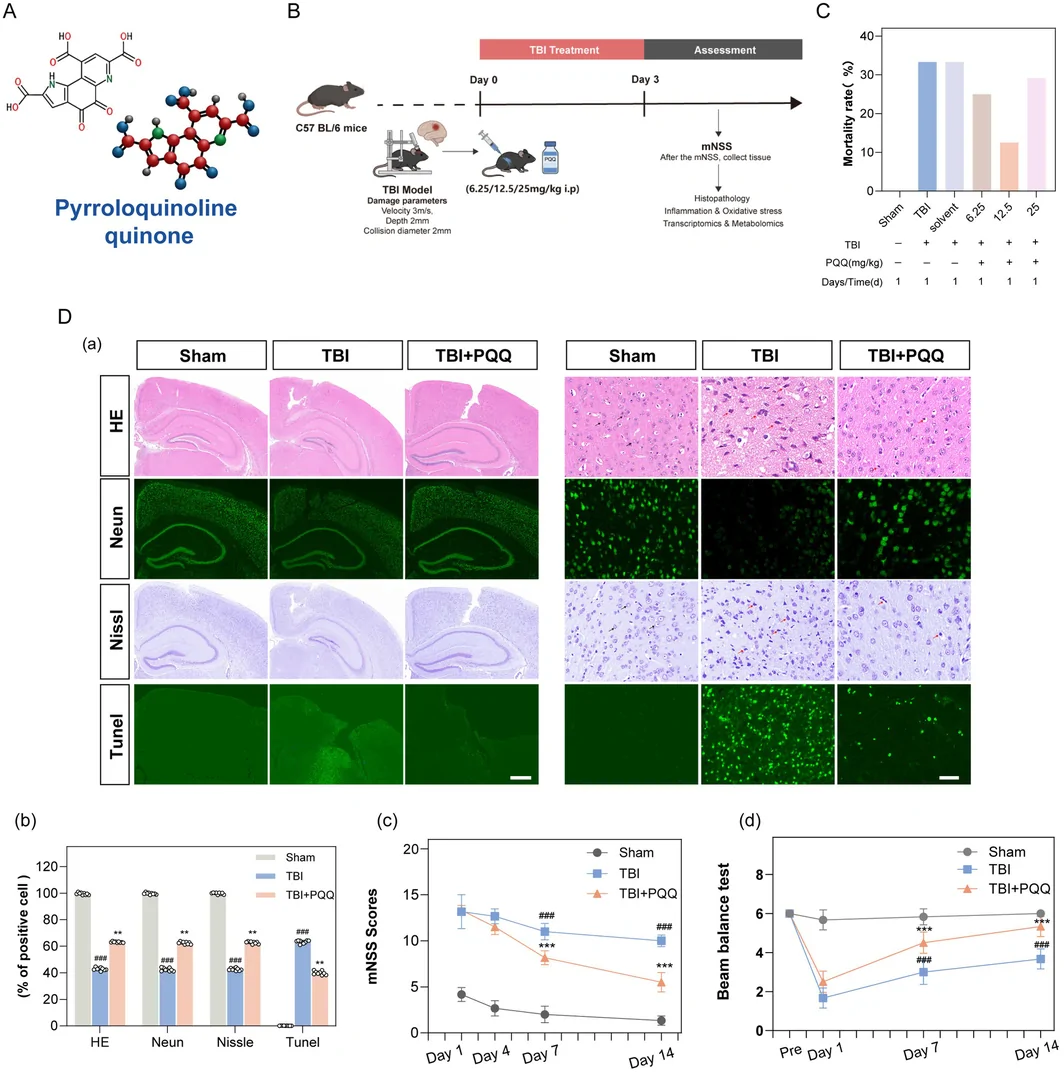
PQQ attenuates TBI‐induced neurological deficits and neuropathological damage. (A) Chemical structure of PQQ. (B) Experimental design. (C) Three‐day mortality (survival cohort, *n* = 24 per group; the corresponding numbers of deaths are given in the Results). (D) (a) Representative HE, NeuN, Nissl and TUNEL images (scale bars: 500 μm and 50 μm). (b) Quantification of positive cell percentages (*n* = 3 per group; three non‐adjacent sections averaged per animal). (c) mNSS scores on days 1, 4, 7 and 14 (Sham, TBI and three PQQ dose groups; *n* = 6 per group). (d) Beam balance scores on days 1, 7 and 14 (Sham, TBI and TBI + PQQ 12.5 mg/kg; *n* = 6 per group). All n values denote independent animals. ###*p < 0.001* versus Sham; **p < 0.01, ***p < 0.001 versus TBI.

C57BL/6 mice received a single i.p. injection of PQQ (6.25, 12.5 or 25 mg/kg) immediately after injury; the structure of PQQ and the study design are shown in Figure 1A,B. In the survival cohort (*n* = 24 per group), 3‐day mortality was 0% in Sham (0/24), 33.3% in TBI (8/24) and 33.3% (8/24) with vehicle, versus 25.0% (6/24) at 6.25 mg/kg, 12.5% (3/24) at 12.5 mg/kg and 29.2% (7/24) at 25 mg/kg (Figure 1C). The dose‐mortality relationship was non‐monotonic, with the greatest reduction at 12.5 mg/kg; the study was not powered for a formal comparison of survival, so these differences are descriptive, and 12.5 mg/kg was used for all mechanistic analyses. TBI produced a marked neurological deficit that PQQ ameliorated dose‐dependently, and the benefit was sustained, with mNSS scores remaining lower than TBI at day 7 and day 14 (Figure 1D,c; ###*p* < 0.001 vs. Sham; ***p < 0.001 vs. TBI); beam balance showed the same pattern, reaching significance at day 7 and becoming more pronounced at day 14 (Figure 1D,d; ***p < 0.001 vs. TBI). Histologically, HE, NeuN, Nissl and TUNEL staining revealed pronounced neuronal loss and apoptosis after TBI, both of which were attenuated by PQQ (Figure 1D,a), and quantification confirmed a dose‐dependent reduction in neuropathological damage (Figure 1D,b; ###*p* < 0.001 vs. Sham; **p < 0.01, ***p < 0.001 vs. TBI).

### 3.2 PQQ Alleviates Neuroinflammation, Brain Edema, and Oxidative Stress After TBI

**FIGURE 2:**
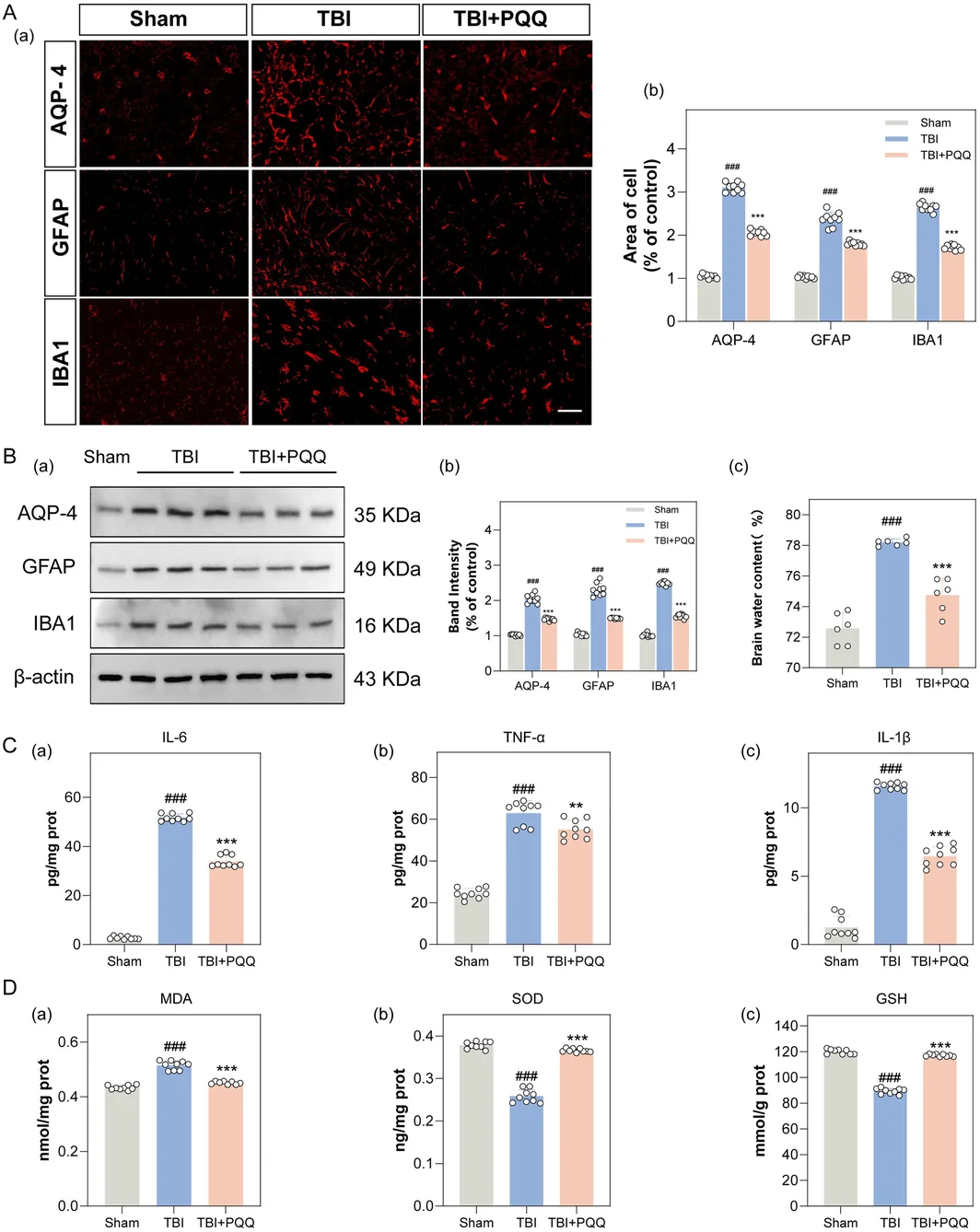
PQQ alleviates neuroinflammation, brain edema, and oxidative stress after TBI. (A) (a) Representative immunofluorescence of AQP‐4, GFAP, and IBA1 (scale bar = 50 μm); (b) quantified positive‐cell areas (% of control; *n* = 3 per group). (B) (a) Representative western blots of AQP‐4, GFAP, IBA1, and β‐Actin; (b) band‐intensity quantification (*n*  = 3 per group); (c) brain water content (%; *n* = 6 per group). (C) ELISA of pro‐inflammatory cytokines: (a) IL‐6, (b) TNF‐α, and (c) IL‐1β (pg/mg protein; *n* = 9 per group). (D) Oxidative‐stress markers: (a) MDA (nmol/mg), (b) SOD (ng/mg), and (c) GSH (mmol/g protein; *n* = 9 per group). Groups: Sham, TBI, TBI + PQQ (12.5 mg/kg). All n values denote independent animals. ###*p* < 0.001 versus Sham; **p < 0.01, ***p < 0.001 versus TBI.

Immunofluorescence revealed elevated AQP‐4, GFAP, and IBA1 expression after TBI, indicative of astrocyte activation, blood–brain barrier disruption, and microglial activation, each of which was attenuated by PQQ (Figure 2A). These alterations were corroborated at the protein level by western blotting, and brain water content was correspondingly lower in the TBI + PQQ group (Figure 2B). In parallel, PQQ suppressed the TBI‐induced elevation of IL‐6, TNF‐α, and IL‐1β (Figure 2C), reduced MDA, and restored SOD and GSH (Figure 2D). Throughout, ###*p* < 0.001 vs. Sham and **p < 0.01, ***p < 0.001 vs. TBI.

### 3.3 Transcriptomic Profiling Reveals Molecular Mechanisms Underlying PQQ Treatment in TBI

**FIGURE 3:**
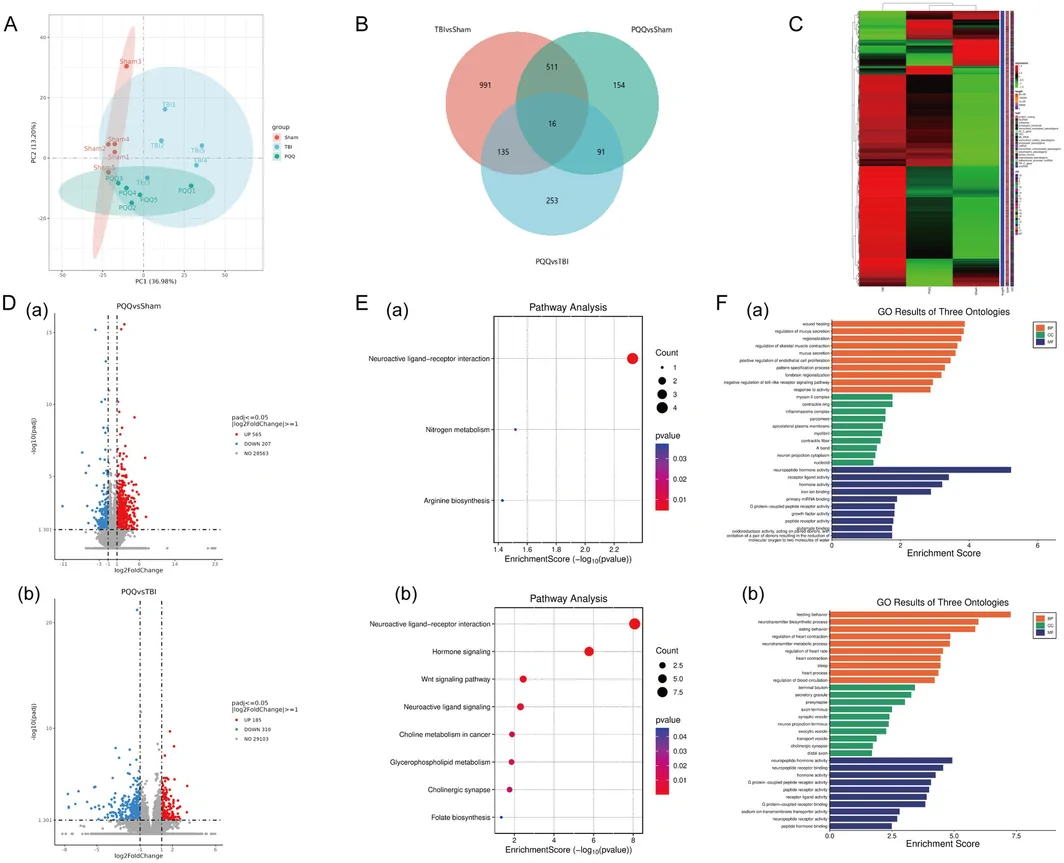
Transcriptomic profiling reveals the molecular mechanisms underlying PQQ treatment in TBI. (A) PCA demonstrating separation of the Sham, TBI and TBI+PQQ groups. (B) Venn diagram of DEG overlap among the three comparisons (TBI vs. Sham, TBI+PQQ vs. Sham, TBI+PQQ vs. TBI). (C) Hierarchical clustering heatmap of DEGs. (D) Volcano plots for (a) TBI+PQQ versus Sham and (b) TBI+PQQ versus TBI; red/blue = up‐/down‐regulated genes. (E) KEGG enrichment for (a) TBI+PQQ versus Sham and (b) TBI+PQQ versus TBI. (F) GO enrichment (BP, MF, CC) for (a) TBI+PQQ versus Sham and (b) TBI+PQQ versus TBI, highlighting arginine biosynthesis and nitrogen metabolism. All panels: *N* = 5 independent mice per group (TBI + PQQ, 12.5 mg/kg).

RNA sequencing of perilesional brain tissue separated the three groups by PCA (Figure 3A); Venn analysis defined the overlap of DEGs across the three comparisons (Figure 3B), and hierarchical clustering revealed group‐specific expression patterns (Figure 3C). Volcano plots delineated the up‐ and down‐regulated genes (Figure 3D), and KEGG enrichment identified arginine biosynthesis and nitrogen metabolism as the most significantly enriched pathways in both comparisons (Figure 3E). Arginine biosynthesis was prioritized on three predefined criteria: it ranked first by adjusted enrichment significance and was enriched in both comparisons; it was the only pathway independently corroborated by both the metabolomic and the network pharmacology analyses; and its core enzymes ASS1 and CPS1 were reproducibly identified as hub targets linking urea‐cycle metabolism to the neuroinflammatory phenotype. Pathways enriched in only one dataset or lacking a plausible mechanistic link, such as nitrogen metabolism, were not prioritized. GO enrichment further highlighted the functional categories altered by PQQ (Figure 3F).

### 3.4 Systems‐Level Analysis Identifies TBI‐Dysregulated Gene Modules Rescued by PQQ

Rescued‐gene analysis demonstrated that PQQ broadly reversed the TBI transcriptional programme: PQQ‐ and TBI‐induced fold‐changes were strongly anticorrelated (Pearson *r* = −0.83), with 98.1% of TBI‐DEGs shifted oppositely and 397 genes classified as PQQ‐rescued, including *Nos2* and *Arg1* (Figure S2A). TBI‐up‐regulated (*n* = 1138) and TBI‐down‐regulated (*n* = 128) genes returned toward the Sham state after PQQ (Figure S2B,C), and the arginine/urea‐cycle genes *Cps1*, *Ass1*, *Slc25a15*, *Gatm*, and *Gls* were significantly reversed (*p* < 0.01; Figure S2D).

Co‐expression analysis of the TBI‐responsive gene set identified an inflammatory module (M1, 1138 genes) and a neuronal module (M2, 127 genes) that were oppositely dysregulated by TBI (M1: Sham *r* = −0.77, TBI *r* = 0.69; M2: Sham *r* = 0.70, TBI *r* = −0.89) but lost significant correlation with group after PQQ (Figure S3A,B). Hub genes included *Lyz2*, *Lcp1*, *Ctsz*, *Cyba*, *Dock2*, and *Ifi30* (M1) and *Nr1d1*, *Per1*, and *Dusp1* (M2) (Figure S3C). Because the analysis was restricted to TBI‐DEGs from five animals per group, M1 and M2 largely recapitulate the up‐ and down‐regulated arms of the TBI signature; they indicate that PQQ normalizes the coordinated transcriptional programme dysregulated by TBI but do not constitute an independent genome‐wide network validation.

### 3.5 Metabolomic Profiling Identifies Key Metabolic Alterations Induced by PQQ in TBI

**FIGURE 4:**
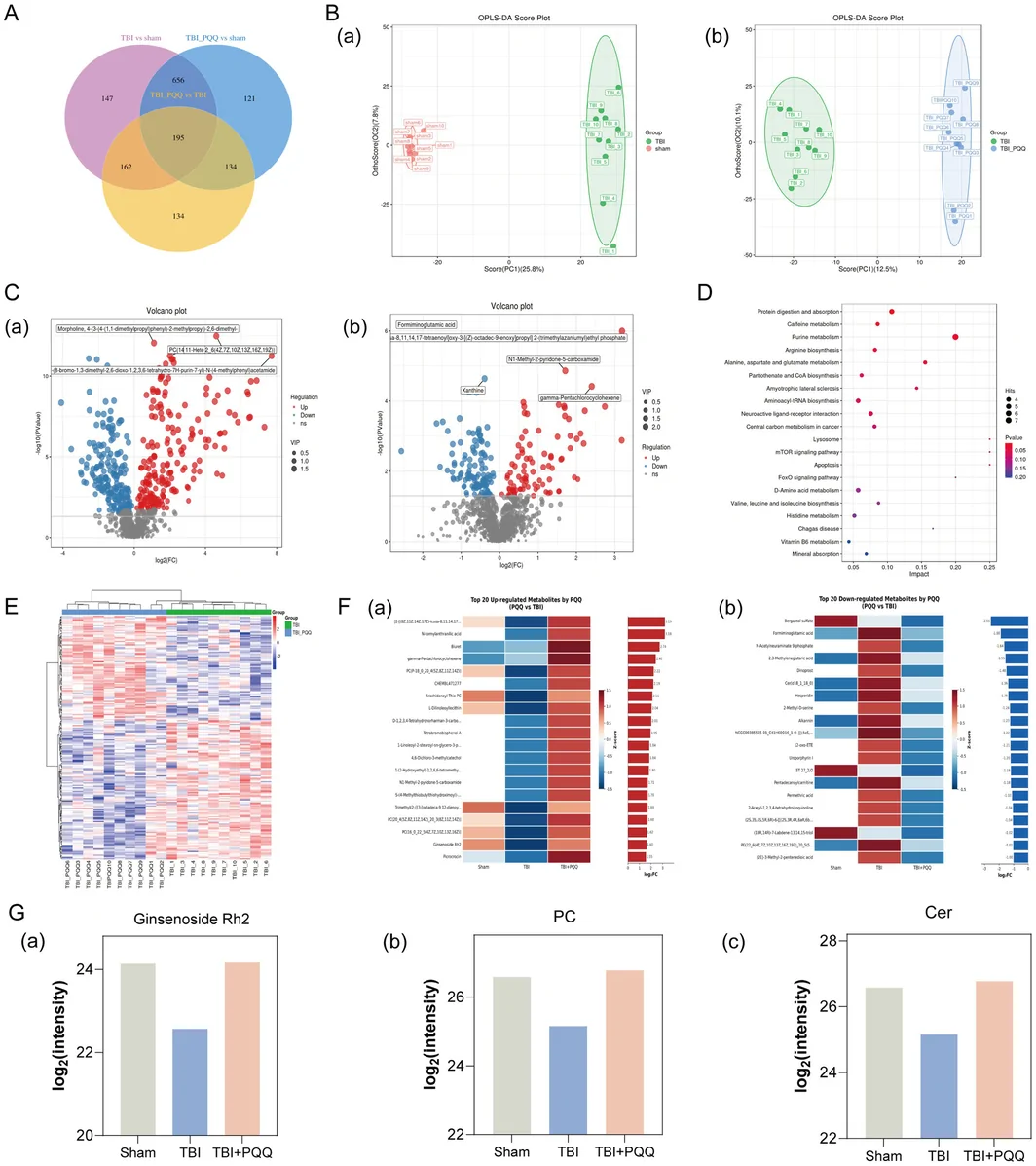
Metabolomic profiling identifies key metabolic alterations induced by PQQ in TBI. (A) Venn diagram of differentially expressed metabolites across three comparisons. (B) OPLS‐DA score plots showing group separation in (a) TBI versus Sham and (b) TBI+PQQ versus TBI. (C) Volcano plots of differentially expressed metabolites with representative metabolites annotated, (a) TBI versus Sham and (b) TBI+PQQ versus TBI. (D) KEGG enrichment bubble plot highlighting arginine biosynthesis and related pathways. (E) Hierarchical clustering heatmap of differentially expressed metabolites. (F) Heatmaps of (a) top 20 up‐ and (b) top 20 down‐regulated metabolites in TBI+PQQ versus TBI. (G) Log_2_ intensities of (a) Ginsenoside Rh2, (b) PC, and (c) Cer. All panels: *N* = 10 independent mice per group (TBI + PQQ, 12.5 mg/kg).

Untargeted metabolomic profiling defined the overlap of differentially expressed metabolites across the three comparisons (Figure 4A), and OPLS‐DA separated TBI from Sham and TBI + PQQ from TBI (Figure 4B). Cross‐validation and permutation testing confirmed that these models were not overfitted (*R* ^2^Y 0.984–0.998, *Q* ^2^ 0.700–0.960; Table S1), and unsupervised PCA with pooled QC samples showed that all replicates fell within the Hotelling's T^2^ 95% confidence ellipse, so no samples were excluded. Volcano plots highlighted the key metabolites (Figure 4C), and KEGG enrichment again identified arginine biosynthesis as the pathway most significantly altered by PQQ (Figure 4D). Hierarchical clustering and the top 20 up‐ and down‐regulated metabolites in the TBI + PQQ versus TBI comparison further characterized this reprogramming (Figure 4E,F), and representative metabolites including ginsenoside Rh2, phosphatidylcholine, and ceramide differed across groups (Figure 4G).

### 3.6 Multi‐Omics Integration Identifies Arginine Biosynthesis as a Key Pathway Regulated by PQQ in TBI

**FIGURE 5:**
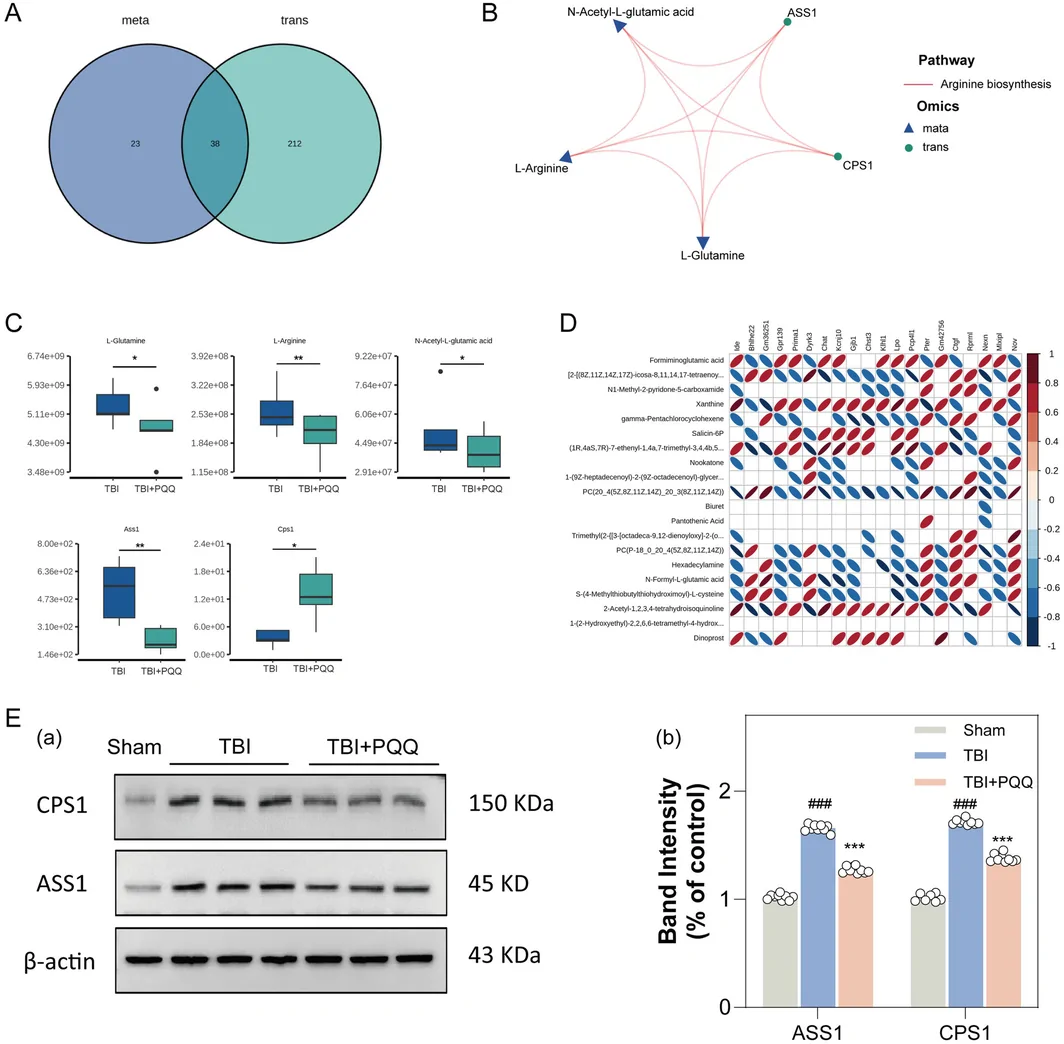
Multi‐omics integration identifies arginine biosynthesis as a key pathway regulated by PQQ in TBI. (A) Venn diagram of overlapping DEMs and DEGs (TBI + PQQ vs. TBI). (B) Arginine biosynthesis network with key nodes ASS1, CPS1, L‐Arginine, L‐Glutamine, and N‐Acetyl‐L‐glutamic acid. (C) Box plots of pathway metabolites and genes (*n* = 5 transcriptomic, *n* = 10 metabolomic per group); *p < 0.05, **p < 0.01. (D) Metabolite–gene correlation heatmap (*n* = 5 transcriptomic, *n* = 10 metabolomic per group). (E) (a) Western blots of CPS1 (150 kDa), ASS1 (45 kDa), and β‐Actin (43 kDa); (b) quantification demonstrating PQQ‐mediated attenuation of TBI‐induced ASS1 and CPS1 protein expression (*n* = 3 per group). ###*p* < 0.001 versus Sham; ***p < 0.001 versus TBI.

Multi‐omics integration delineated the overlap between differentially expressed metabolites and genes in the TBI + PQQ versus TBI comparison (Figure 5A), and a network of the arginine biosynthesis pathway integrating both datasets highlighted ASS1, CPS1, L‐arginine, L‐glutamine and N‐acetyl‐L‐glutamic acid as central nodes (Figure 5B). These metabolites and the genes ASS1 and CPS1 were significantly altered in TBI + PQQ versus TBI animals (*p < 0.05; **p < 0.01; Figure 5C), with significant associations between metabolites and hub genes (Figure 5D). Western blotting further confirmed that PQQ attenuated the TBI‐induced elevation of ASS1 (45 kDa) and CPS1 (150 kDa) protein (Figure 5E; ###*p* < 0.001 vs. Sham; ***p < 0.001 vs. TBI). Arginine itself was not quantified, and citrulline, ornithine, nitric oxide and iNOS activity were not measured, so these data identify ASS1/CPS1‐driven arginine biosynthesis as a pathway modulated by PQQ rather than as a validated mechanism.

### 3.7 Network Pharmacology and Molecular Docking Predict ASS1 and CPS1 as Candidate Molecular Targets of PQQ in TBI

Integration of PQQ‐related targets from CTD, TargetNet and STITCH with TBI‐related targets from GeneCards and OMIM identified 59 overlapping targets (Figure S1A), whose protein–protein interaction network revealed ASS1, CPS1, CASP3 and MAPK1 as prominent hub nodes (Figure S1B); KEGG enrichment highlighted arginine biosynthesis, nitrogen metabolism and pathways of neurodegeneration (Figure S1C). Four centrality algorithms consistently identified ASS1 and CPS1 as shared core targets (Figure S1D), and docking predicted favorable binding interactions between PQQ and both enzymes (Figure S1E). These results suggest ASS1 and CPS1 as candidate molecular targets of PQQ in TBI; biochemical validation (e.g., SPR, ITC, CETSA, DARTS or enzyme activity assays) would be required to confirm direct physical interaction.

### 3.8 PQQ Promotes PINK1/Parkin‐Mediated Mitophagy and Restores Mitochondrial Function After TBI

**FIGURE 6:**
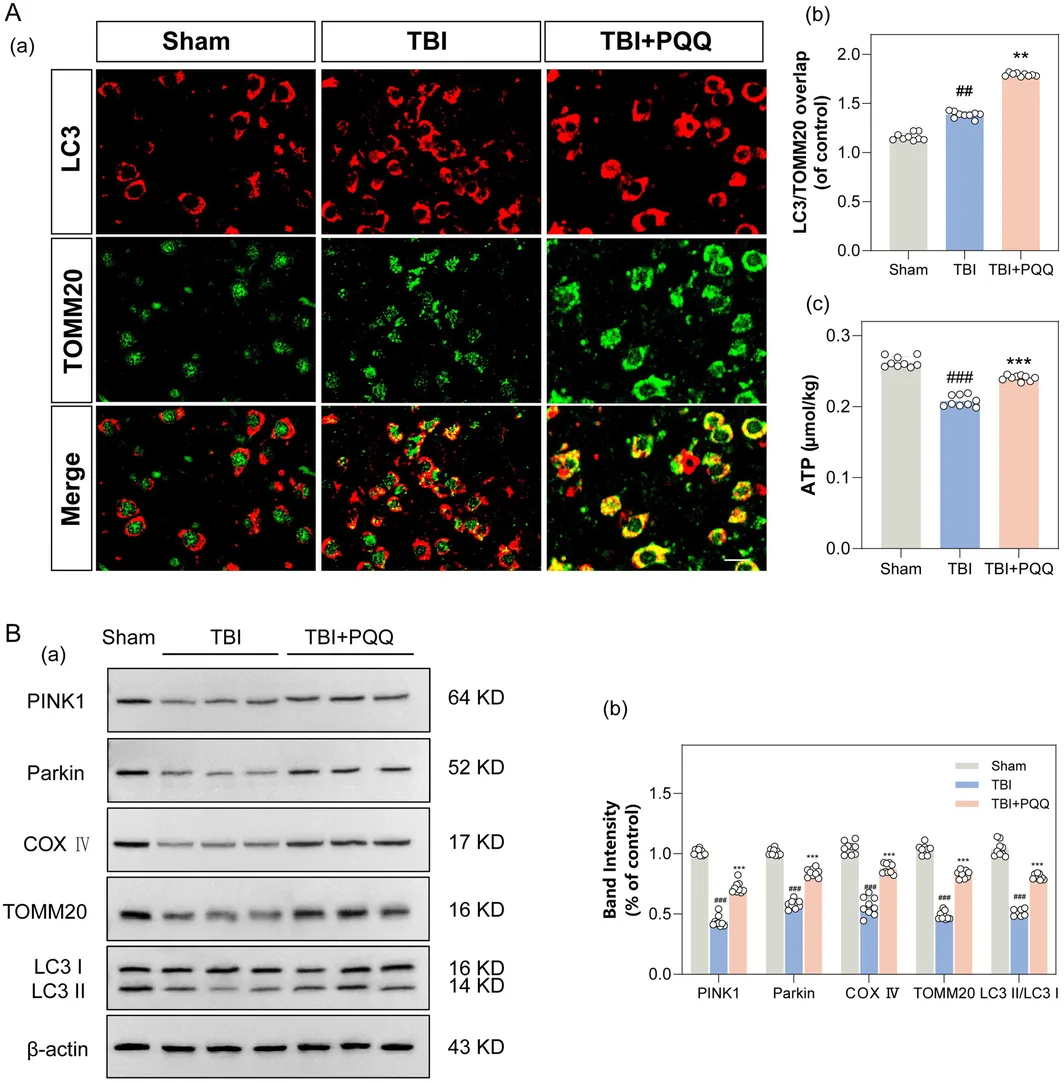
PQQ promotes PINK1/Parkin‐mediated mitophagy and restores mitochondrial function after TBI. (A) (a) Confocal immunofluorescence of LC3 (red) and TOMM20 (green) co‐localisation (scale bar = 20 μm); (b) LC3/TOMM20 overlap quantification (% of control; *n* = 3 per group); (c) ATP levels (μmol/kg; *n* = 9 per group). ###*p* < 0.001 versus Sham; **p < 0.01, ***p < 0.001 versus TBI. (B) (a) Western blots of PINK1 (64 kDa), Parkin (52 kDa), COX IV (17 kDa), TOMM20 (16 kDa), LC3‐II/LC3‐I (14/16 kDa), and β‐Actin (43 kDa); (b) band‐intensity quantification (% of control; *n* = 3 per group). ##*p* < 0.01 versus Sham; **p < 0.01, ***p < 0.001 versus TBI. All n values denote independent animals.

Confocal imaging demonstrated greater LC3 (red)/TOMM20 (green) co‐localisation in the TBI + PQQ group than in the TBI group (Figure 6A; ###*p* < 0.001 vs. Sham; **p < 0.01, ***p < 0.001 vs. TBI), and ATP levels were restored by PQQ (Figure 6A,c). Western blotting further showed that PQQ increased PINK1 (64 kDa), Parkin (52 kDa), COX IV (17 kDa) and TOMM20 (16 kDa) expression and elevated the LC3‐II/LC3‐I ratio while reducing LC3‐I (Figure 6B; ##*p* < 0.01 vs. Sham; **p < 0.01, ***p < 0.001 vs. TBI). Because flux markers (p62/SQSTM1, BNIP3, NIX, OPTN) and LC3 turnover assays were not employed, these indices cannot formally distinguish enhanced mitophagy initiation from blocked downstream degradation.

## 4 Discussion

In the present study, PQQ attenuated neurological deficits and secondary brain injury in a murine CCI model, accompanied by changes in markers of PINK1/Parkin‐mediated mitophagy initiation, rather than confirmed restoration of mitophagic flux and suppression of ASS1/CPS1‐driven arginine biosynthesis. The neuroprotective potential of PQQ in TBI has been reported previously, although the molecular mechanisms were not examined in detail [13]. By combining transcriptomic and metabolomic profiling with network pharmacology and docking, the present work describes two processes concurrently modulated by PQQ: impaired mitochondrial quality control and aberrant urea‐cycle‐driven neuroinflammation. Because loss‐of‐function experiments were not performed, these relationships are hypothesis‐generating rather than causal.

The secondary injury cascade is driven by interconnected mitochondrial dysfunction, oxidative stress, and neuroinflammation, in which mitochondrial metabolic reprogramming contributes to an immunometabolic axis governing microglial activation [14]; this has motivated interest in agents engaging more than one node. Phytochemicals such as curcumin and resveratrol act largely through antioxidant pathways, but limited bioavailability and single‐axis mechanisms constrain their efficacy [15]. Among mitochondria‐targeting strategies under investigation for TBI, triphenylphosphonium‐conjugated antioxidants such as MitoQ and SkQ1 scavenge matrix ROS but act principally on the oxidative arm [16]; elamipretide stabilizes cristae architecture and has reached clinical testing in mitochondrial myopathies [17]; cyclosporin A inhibits the permeability transition pore, but its benefit in TBI is inconsistent and immunosuppression limits its use [18]; and NAD^+^ precursors act upstream on sirtuin signaling and mitochondrial biogenesis [19]. PQQ is distinguished less by potency than by profile: it is orally bioavailable, is a dietary compound rather than a synthetic agent, and was here associated with changes in both mitophagy and urea‐cycle metabolism [20]. It has not, however, been compared head‐to‐head with these agents in TBI, so no claim of superiority is warranted.

Transcriptomic analysis identified ASS1 among the most significantly up‐regulated genes in TBI versus Sham, with arginine biosynthesis the top‐ranked KEGG pathway. This is consistent with transcriptional activation of the urea cycle after TBI, which may replenish arginine pools that sustain iNOS‐driven nitric oxide production and amplify neuroinflammatory signaling in activated microglia [21]. Although CPS1 mRNA was reduced in TBI, CPS1 protein was markedly up‐regulated and was subsequently suppressed by PQQ, a pattern that probably reflects post‐transcriptional and post‐translational regulation, including allosteric control by N‐acetylglutamate and ubiquitin‐proteasome‐mediated turnover, which can decouple mRNA abundance from protein levels [22]. Beyond its canonical urea‐cycle function, ASS1 sustains iNOS activity by depleting intracellular citrulline through the citrulline‐NO cycle, giving it a dual metabolic and inflammatory role [23]. The PQQ‐associated suppression of ASS1 and CPS1 protein is consistent with disruption of this feedforward axis and may contribute to the observed reductions in IL‐6, TNF‐α, and IL‐1β.

Under physiological conditions PINK1 is imported into intact mitochondria and rapidly cleaved by the presenilin‐associated rhomboid‐like protease, keeping basal levels low; only when membrane potential collapses does PINK1 stabilize on the outer membrane, recruit Parkin and initiate mitophagic clearance [24]. In the present model, PINK1 and Parkin protein levels were reduced relative to Sham on day 3. This decline may indicate that the clearance machinery was overwhelmed by the surge of depolarised mitochondria rather than an absence of mitophagic demand [25]; the concomitant reductions in TOMM20 and COX IV are in keeping with net mitochondrial loss. PQQ treatment increased PINK1 and Parkin expression and elevated both the LC3‐II/LC3‐I ratio and LC3/TOMM20 co‐localisation, changes that are indicative of augmented initiation of the mitophagic programme. It should nevertheless be emphasized that these indices are steady‐state measures of autophagosome abundance. Consequently, they cannot discriminate between two mutually exclusive interpretations: PQQ may have promoted autophagosome biogenesis and mitochondrial sequestration, or it may have impaired lysosomal degradation and thereby produced autophagosome accumulation. Because both scenarios generate an identical marker profile, the present data cannot resolve this ambiguity. Definitive interpretation therefore requires direct interrogation of mitophagic flux, for example by bafilomycin A1‐based LC3 turnover assays or mt‐Keima reporters, which would establish whether PQQ genuinely accelerates mitophagic clearance rather than obstructing downstream degradation.

Mitophagy and arginine metabolism may form a mutually reinforcing loop after TBI: damaged mitochondria accumulate, deficient PINK1/Parkin signaling fails to clear them, and the resulting ROS sustain microglial activation, while the urea cycle is activated through the N‐acetylglutamate synthase/CPS1/ASS1 cascade, expanding the arginine pool available for iNOS‐driven neuroinflammation. The present data are compatible with PQQ acting upon both arms of this loop. Whether PQQ interrupts this amplification at two mechanistically coupled nodes, and whether the arms are causally linked here, cannot be resolved from observational data; the mitophagy‐arginine axis is therefore nominated as a candidate target pending causal validation.

This study has several limitations. Although neurological function was monitored to day 14, the histological, biochemical and multi‐omics endpoints were assessed only at the acute 3‐day phase, so the temporal dynamics of the two mechanistic arms and long‐term cognitive outcomes remain undefined. Causal validation of ASS1 and CPS1 as PQQ targets by knockdown or knockout was not undertaken, and the docking results remain computational predictions that await biochemical confirmation. Critically, the proposed dual PINK1/Parkin and ASS1/CPS1 axis is largely built upon correlative observations, and the establishment of causality will require dedicated loss‐of‐function studies. Two complementary strategies are available. The first is genetic, comprising siRNA‐ or shRNA‐mediated knockdown and conditional knockout of PINK1, Parkin, ASS1 or CPS1. The second is pharmacological, comprising inhibition of mitophagy or of PINK1/Parkin signaling together with inhibition of ASS1/CPS1 enzymatic activity. Each approach should be coupled with target‐engagement assays. Until such interventional data become available, the mechanism proposed here should be regarded as a candidate hypothesis rather than an experimentally established pathway. Several metabolic readouts were likewise not obtained: arginine itself was not quantified, and citrulline, ornithine, nitric oxide and iNOS activity were not determined, so the downstream metabolic consequences of ASS1/CPS1 suppression remain inferential. Similarly, mitophagy was inferred from LC3/TOMM20 co‐localisation, the LC3‐II/LC3‐I ratio and PINK1/Parkin expression. Because these indices report steady‐state autophagosome abundance rather than completed mitophagic flux, enhanced mitophagy initiation cannot be formally distinguished from impaired lysosomal degradation with secondary autophagosome accumulation. Only male C57BL/6 mice were studied, to avoid the confounding effect of oestrous‐cycle variation on injury severity and on the metabolomic profile; since estrogen and progesterone are neuroprotective and attenuate microglial activation [26], and sex differences exist in mitochondrial bioenergetics, mitophagy regulation and urea‐cycle enzyme expression, both mechanistic arms could behave differently in females. Confirmation in female animals is therefore essential before these findings can be generalized across sexes or advanced toward clinical translation. Finally, PQQ is well tolerated in human trials in other indications [7, 20], but no clinical data exist in TBI. Because PQQ was administered immediately after injury, the present findings establish early neuroprotective potential but do not address the delay with which most patients reach clinical care; future studies should test whether delayed administration remains effective and define how far the therapeutic window extends. Future work should extend the observation window, include both sexes, test clinically realistic delayed treatment windows, add loss‐of‐function and target‐engagement experiments, and evaluate PQQ in larger preclinical models.

## 5 Conclusion

PQQ attenuated secondary brain injury and improved neurological outcome after TBI, in association with increased markers of PINK1/Parkin‐mediated mitophagy initiation and suppressed ASS1/CPS1‐driven arginine biosynthesis. These findings identify the mitophagy‐arginine axis as a candidate dual‐axis target and support further evaluation of PQQ as a neuroprotective agent for TBI.

## Author Contributions

Y.H.: Investigation, methodology, formal analysis, writing – original draft. L.Y.: Investigation, methodology, formal analysis, writing – original draft. Y.L.: Investigation, formal analysis, writing – original draft. Z.Z.: Investigation, validation. A.L.: Investigation, validation. W.M.: Conceptualization, supervision, writing – review and editing. Y.M.: Conceptualization, supervision, project administration, writing – review and editing.

## Funding

The authors have nothing to report.

## Ethics Statement

All animal experiments were approved by the Institutional Animal Care and Use Committee (IACUC) of the First Medical Center of Chinese PLA General Hospital (Approval No.: 2026‐X23‐28) and conducted in strict compliance with the National Institutes of Health (NIH) Guide for the Care and Use of Laboratory Animals (8th edition, 2011) and the ARRIVE guidelines 2.0. All efforts were made to minimize animal suffering and reduce the number of animals used in this study.

## Conflicts of Interest

The authors declare no conflicts of interest.

## Supporting information


**Figure S1:** Network pharmacology and molecular docking predict ASS1 and CPS1 as candidate molecular targets of PQQ in TBI. (A) Venn diagram of 59 overlapping PQQ/TBI targets with UpSet plot. (B) PPI network; hub nodes ASS1, CPS1, CASP3, and MAPK1 highlighted (red). (C) KEGG enrichment highlighting arginine biosynthesis, nitrogen metabolism, and neurodegeneration. (D) Four centrality algorithms (MCC, EPC, Degree, MNC) and Venn diagram confirm ASS1 and CPS1 as shared hubs. (E) Docking poses and 2D interaction diagrams of PQQ with (a) ASS1 and (b) CPS1. All panels in silico; panel C cross‐referenced with transcriptomic data (*n* = 5 mice/group).

**Figure S2:** PQQ restores the TBI‐dysregulated transcriptome (rescued‐gene analysis). (A) log2FC scatter plot of TBI‐DEGs (TBI + PQQ vs. TBI versus TBI vs. Sham); 397 rescued genes highlighted, including Nos2 and Arg1 (Pearson r = −0.83; 98.1% reversed). (B) Row z‐score heatmap of 1266 TBI‐DEGs. (C) Rescue trajectories of TBI‐up (*n* = 1138) and TBI‐down (*n* = 128) gene sets. (D) Arginine/urea‐cycle gene expression across groups (*p < 0.05, **p < 0.01, PQQ vs. TBI). *n* = 5 independent mice per group.

**Figure S3:** Co‐expression modules within the TBI‐responsive gene set are dysregulated by TBI and normalized by PQQ. (A) Module‐group Pearson correlations for inflammatory module M1 (1138 genes) and neuronal module M2 (127 genes), from WGCNA restricted to 1266 TBI‐DEGs. (B) Module eigengene trajectories showing TBI dysregulation and PQQ normalization of M1 and M2. (C) Hub genes ranked by module membership (kME). *n* = 5 independent mice per group.

**Table S1:** Model validation parameters of the OPLS‐DA models used in the untargeted metabolomic analysis.

## Data Availability

The data that support the findings of this study are available on request from the corresponding author. The data are not publicly available due to privacy or ethical restrictions.
