# Supplementary material for: Pyrroloquinoline Quinone Attenuates Traumatic Brain Injury‐Induced Secondary Damage by Activating PINK1/Parkin‐Mediated Mitophagy and Suppressing ASS1/CPS1‐Driven Arginine Biosynthesis
Source: CNS Neurosci Ther. 2026 Sep 9;32(9):e71146. doi: 10.1002/cns.71146 (PMC13559014; doi:10.1002/cns.71146)
Supplement: Supplementary file 1 [file CNS-32-e71146-s001.docx]

**Table S1.** Model validation parameters of the OPLS-DA models used in the untargeted metabolomic analysis.

| **Comparison** | **Ion mode** | **Predictive components** | **R^2X^** | **R^2Y^** | **Q^2^** | **Permutations** | **Overfitting** |
| --- | --- | --- | --- | --- | --- | --- | --- |
| TBI vs. Sham | Positive | 1 + 1 + 0 | 0.337 | 0.996 | 0.937 | 100 | No |
| TBI vs. Sham | Negative | 1 + 1 + 0 | 0.361 | 0.994 | 0.927 | 100 | No |
| TBI+PQQ vs. Sham | Positive | 1 + 1 + 0 | 0.312 | 0.998 | 0.953 | 100 | No |
| TBI+PQQ vs. Sham | Negative | 1 + 1 + 0 | 0.364 | 0.997 | 0.960 | 100 | No |
| TBI+PQQ vs. TBI | Positive | 1 + 1 + 0 | 0.225 | 0.986 | 0.700 | 100 | No |
| TBI+PQQ vs. TBI | Negative | 1 + 1 + 0 | 0.220 | 0.984 | 0.718 | 100 | No |
